# Differentially expressed and survival‐related proteins of lung adenocarcinoma with bone metastasis

**DOI:** 10.1002/cam4.1363

**Published:** 2018-03-09

**Authors:** Mengdi Yang, Yi Sun, Jing Sun, Zhiyu Wang, Yiyi Zhou, Guangyu Yao, Yifeng Gu, Huizhen Zhang, Hui Zhao

**Affiliations:** ^1^ Department of Internal Oncology Shanghai Sixth People's Hospital Affiliated to Shanghai Jiaotong University Shanghai 200233 China; ^2^ Department of Radiology Shanghai Sixth People's Hospital Affiliated to Shanghai Jiaotong University Shanghai 200233 China; ^3^ Department of Internal Oncology Shanghai Sixth People's Hospital Soochow University Shanghai 200233 China; ^4^ Department of Pathology Shanghai Sixth People's Hospital Affiliated to Shanghai Jiaotong University Shanghai 200233 China

**Keywords:** Bone metastasis, lung adenocarcinoma, proteomics, survival, the cancer genome atlas

## Abstract

Despite recent advances in targeted and immune‐based therapies, the poor prognosis of lung adenocarcinoma (LUAD) with bone metastasis (BM) remains a challenge. First, two‐dimensional gel electrophoresis (2‐DE) was used to identify proteins that were differentially expressed in LUAD with BM, and then matrix‐assisted laser desorption/ionization time of flight mass spectrometry (MALDI‐TOF‐MS) was used to identify these proteins. Second, the Cancer Genome Atlas (TCGA) was used to identify mutations in these differentially expressed proteins and Kaplan–Meier plotter (KM Plotter) was used to generate survival curves for the analyzed cases. Immunohistochemistry (IHC) was used to check the expression of proteins in 28 patients with BM and nine patients with LUAD. Lastly, the results were analyzed with respect to clinical features and patient's follow‐up. We identified a number of matched proteins from 2‐DE. High expression of enolase 1 (ENO1) (HR = 1.67, logrank *P* = 1.9E‐05), ribosomal protein lateral stalk subunit P2 (RPLP2) (HR = 1.77, logrank *P* = 2.9e‐06), and NME/NM23 nucleoside diphosphate kinase 2 (NME1‐NME2) (HR = 2.65, logrank *P* = 3.9E‐15) was all significantly associated with poor survival (*P *<* *0.05). Further, *ENO1* was upregulated (*P *=* *0.0004) and calcyphosine (*CAPS1*) was downregulated (*P *=* *5.34E‐07) in TCGA LUAD RNA‐seq expression data. IHC revealed that prominent ENO1 staining (OR = 7.5, *P *=* *0.034) and low levels of CAPS1 (OR = 0.01, *P *<* *0.0001) staining were associated with BM incidence. Finally, we found that LUAD patients with high expression of ENO1 and RPLP2 had worse overall survival. This is the first instance where the genes *ENO1*,* RPLP2*,* NME1‐NME2* and *CAPS1* were associated with disease severity and progression in LUAD patients with BM. Thus, with this study, we have identified potential biomarkers and therapeutic targets for this disease.

## Introduction

Lung cancer is the deadliest type of cancer in both men and women, with lung adenocarcinoma (LUAD) as the most common subtype of this disease 1, 2. As with other tumors, bones are a suitable substrate for tumor metastases because bone matrix contains high concentrations of numerous growth factors that may stimulate the proliferation of tumor cells. Bone metastases (BM) can lead to skeletal‐related events (SREs), such as pathologic fractures, spinal cord or nerve compression, and hypercalcemia. Further, BM can complicate patient treatment by necessitating targeted radiation or bone surgery 3. The presence of BM not only shortens the overall survival (OS) of these patients (median 6–12 months) 4, but also results in a significantly increased financial burden for patients and the healthcare system overall 5. Although primary bone tumors and BM have been extensively studied, the molecular differences between LUAD and BM remain largely unstudied.

The pathogenesis of metastasis at the systemic, cellular, and molecular levels are important areas of cancer research. Proteomics bridges the gap between genomic information and functional biology and can serve to identify new insights into this disease 6. Oncoproteomics is the study of proteins and their interactions in a cancer network. Recent studies have indicated that the existence of intratumor heterogeneity in cancer 7. The Cancer Genome Atlas (TCGA) data portal (http://cancergenome.nih.gov/) is the largest and most commonly used public resource for cancer genomics, providing data from thousands of tumor samples 8, and these data have presented a new challenge of explaining how genomic alterations drive cancers 9.

In this study, we used proteomics approaches and publically available data from TCGA to identify proteins that are differentially expressed and associated with significant differences in survival in LUAD patients with BM.

## Materials and Methods

### Clinical samples

Five patients with traumatic amputation, five patients with bone infiltration, nine patients with LUAD who underwent curative resection or thoracoscopic lobectomy, and 32 LUAD patients with BM who underwent bone biopsy in the Shanghai Sixth People's Hospital (China) from May 2014 to January 2017 were included in this study. All research was in accordance with the tenets of the Declaration of Helsinki followed by informed consent from each subject. These studies were approved by the Ethics Committee of Shanghai Sixth People's Hospital, Shanghai Jiao Tong University. All tumors were reclassified according to the World Health Organization classification of lung cancer for the year 2015 10. Three groups of bone tissues were used in our proteomics study, normal bone, bone infiltration, LUAD with BM, respectively, and each group included five samples. A total of 28 bone biopsy samples and nine LUAD samples were used for immunohistochemistry assays (IHC) and OS (follow‐up period of more than 1 year). Four bone biopsy samples, four LUAD, and para‐LUAD samples were randomly selected for Western blot (WB) analyses. Criteria for enrollment were as follows: (1) a histopathologic diagnosis of LUAD, (2) no history of other tumors, (3) the availability of sufficient tumor sample, and (4) the potential for the patient to participate in follow‐up studies. The main characteristics of TCGA are provided in Table 1. The clinical features of participating patients are listed in Table 2.

**Table 1:** Clinical characteristics of LUAD in TCGA

Clinical features	TCGA				
	Case	Mean	SD	Median	Percent (%)
Age	522	65.33	1.079	66	26.20
<60	139	52.77	5.820	54	26.24
≥60	364	70.13	6.514	70	69.69
Not available	19				3.67
Gender	522				
Men	242				46.40
Women	280				53.60
Clinical stage					
I	279				53.45
II	124				23.75
III	85				16.28
IV	26				4.98
TNM stage					
Tumor size					
T1	172				33.01
T2	281				53.67
T3	47				9.07
T4	19				3.47
TX	3				0.58
Lymph node					
N0	335				64.18
N1	98				18.77
N2	75				14.37
N3	2				0.003
NX	12				0.023
Distant metastasis					
M0	353				67.62
M1	25				4.79
MX	144				27.59

TCGA, the Cancer Genome Atlas; SD, standard deviation. *P *<* *0.05 was considered statistically significant.

**Table 2:** Baseline characteristics for all patients enrolled in the study

Characteristic	Proteomics			*P*	Other		*P*
	Bone	LUAD	BM		LUAD	BM	
Sex							
Male	3	1	2	0.8	5	19	0.691
Female	2	4	3		4	9	
Age							
Mean ± SD	33 ± 6.82	61.40 ± 9.42	61.20 ± 12.46		57.56 ± 9.74	61.32 ± 9.05	0.78
TNM							
T1		2			4	2	
T2		3	1		4	5	
T3			2			12	
T4			2		1	4	
TX						5	
N0					5	4	
N1		2	2		1	7	
N2		1	2		3	9	
N3			1			4	
NX						4	
M0					9		
M1			5			28	
MX							
Stage							
I		2			5		
II		1			1		
III		2			3		
IV			5			28	
Smoker							
Yes	3	2	4	0.8	4	20	0.229
No	2	3	1		5	8	

LUAD, lung adenocarcinoma; BM, bone metastasis; SD, standard deviation. Other means other study which included the following: imaging information, immunohistochemistry, and Western blot study. *P *<* *0.05 was considered statistically significant.

### Proteomics study

Protein extraction: samples were washed with normal saline, cut into 1 mm^3^ pieces (~300 mg each), ground in liquid nitrogen, and then lysed in 1 mL lysis buffer (4 mol/L urea, 2 mol/L thiourea, 4% CHAPS, 0.2% carrier ampholyte (3‐10NL), cocktail (Roche)). Next, samples were homogenized using a DOUNCE homogenizer, transferred to a centrifuge tube, and then sonicated (80 W, 10 sec for eight times, 15 sec apart, then placed on ice). The whole process was carried out in an ice bath. Lysates were then clarified by centrifugation at 18407 g for 1 h, and then, the supernatant was collected. Bio‐Rad protein assay reagent was used to quantify the protein concentration of each sample, which were then divided into fractions of 100 *μ*g protein in individual 500‐*μ*L centrifuge tubes, and frozen at −80°C. For two‐dimensional electrophoresis (2‐DE), we loaded 100 *μ*g samples into each lane of a 2‐DE gel, with IEF of pH3‐10 on nonlinear strips (Amersham). 2‐DE was then run at 30 V for 12 h, 500 V for 1 h, 1000 V for 1 h, 8000 V for 8 h, and 500 V 4 h. Gels were dyed with silver staining, then scanned on a flatbed scanner, and analyzed with Adobe Photoshop. Protein spots that were judged to be differentially expressed between LUAD primary tumors and BM were then cut from the gel, digested, and analyzed by MALDI‐TOF proteomics. Protein spots exhibiting at least a 1.3‐fold change and that were statistically significant (*t*‐test <0.05) were considered as differentially expressed.

### The cancer genome atlas

We downloaded RNA‐seq and clinical data from publically available TCGA datasets to further verify differentially expressed genes using the “DESeq” package and explored their relationship with survival using the “survival” package in R.

### KM plotter

Kaplan–Meier survival plots, hazard ratios, and logrank *P* were calculated in KM Plotter (http://kmplot.com). The clinical characteristics can be found on this website. Gene expression data and relapse‐free and overall survival information derived from the GEO (Affymetrix microarrays only), EGA, and TCGA databases, which integrates gene expression and clinical data simultaneously via a PostgreSQL server. The patient samples are divided into two groups to assess the prognostic value of a particular gene based on the various quantile expressions of the proposed biomarker. Then, a Kaplan–Meier survival plot is generated by the two patient cohorts, and the hazard ratio (HR) with 95% confidence intervals (CI) and logrank *P* value are calculated. Each database is updated biannually.

### Western blot assay

Western blot, LUAD, and para‐LUAD tissue were prepared with RIPA buffer. Equal amounts of protein were loaded onto SDS‐PAGE gels, separated by electrophoresis, transferred onto a polyvinylidene fluoride membrane, and incubated with primary antibodies against ENO1 (Abcam, UK). A horseradish peroxidase‐conjugated secondary antibody (Jackson ImmunoResearch, USA) was used, and blots were developed with the ECL Plus reagent (Millipore, Burlington, MA).

### Immunohistochemical assays

After antigen retrieval at high pH for 20 min, paraffin‐embedded sections were incubated with primary antibodies against enolase 1 (ENO1) (Abcam), ribosomal protein lateral stalk subunit P2 (RPLP2) (Abcam), calcyphosine (CAPS1) (Abcam), NME/NM23 nucleoside diphosphate kinase 2 (NMEI‐NME2) (NOVA, Beaverton, OR), respectively, washed, nd then incubated with biotinylated secondary antibody (Kirkegaard & Perry Laboratories, Gaithersburg, MD). Fromowitz's standard was used to semiquantitatively assess the staining of these proteins 11. The minimum score was 0 which meant negative; otherwise, it is positive. We define three points as cutoff point. 0–3 points as low expression, 4–7 as high expression. The diagnoses were confirmed by at least two certified pathologists who were blind to the patients’ information.

### Statistical analysis

Comparisons between expression of proteins were analyzed with SPSS version 20.0 (SPSS Inc, Chicago, IL,) using chi‐square tests (Fisher's Exact Test). Kaplan–Meier survival analyses were used to determine the correlation between selected parameters and OS. The characteristic of enrolled patients was analyzed by independent sample *t*‐test, nonparametric tests, and Fisher's exact test. All tests were two sided. All data were presented as the mean ± SD. Alpha (the probability of a Type I error) for all statistical tests is 0.05. *P*‐value <0.05 was considered statistically significant.

## Results

### Patient image features

**Figure 1:**
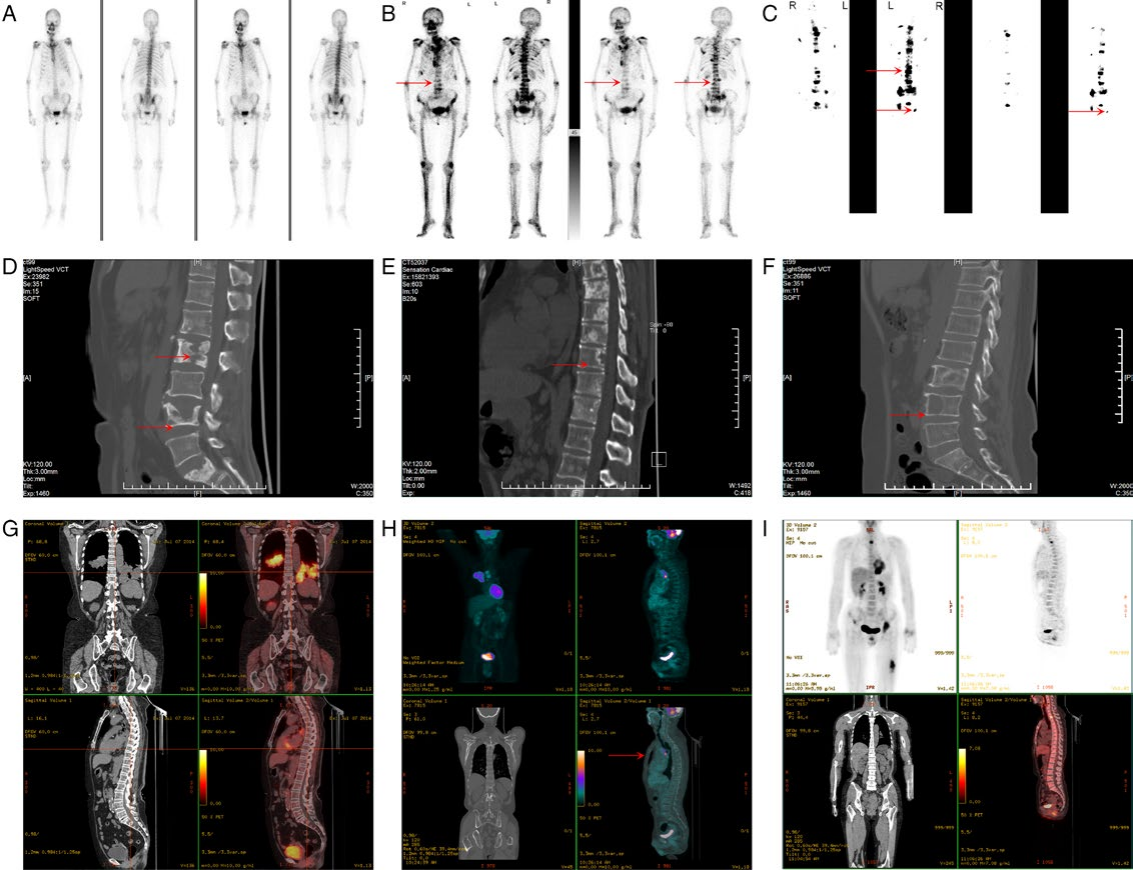
Imaging features of LUAD with BM. (A) ECT from LUAD without BM. (B–C) ECT from LUAD with BM. (D‐F) CT from LUAD with BM. (G‐H) PET‐CT from LUAD without BM. (I) PET‐CT from LUAD with BM. Arrows indicate the focus of BM (arrows).

We collected 37 patients (28 LUAD with BM and nine LUAD) whose disease burden were verified by emission computed tomography (ECT) (Fig. 1A–C), computed tomography (CT) (Fig. 1D–F), and positron emission tomography (PET‐CT) (Fig. 1G–I) and considered a positive bone biopsy as the gold standard for BM.

### Proteomics study

**Figure 2:**
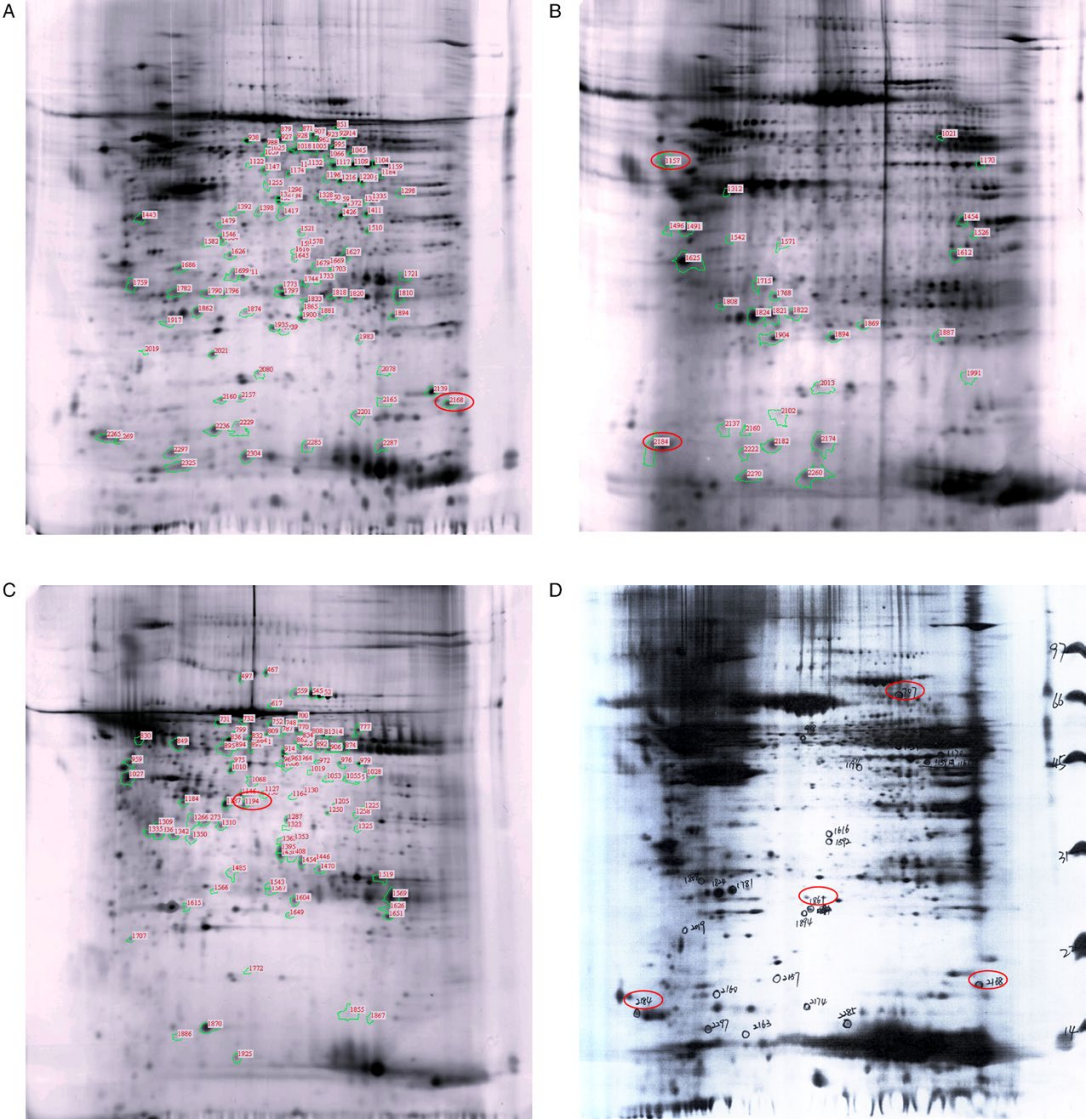
Proteomics of LUAD with BM. (A) The LUAD with BM patients. (B) Bone infiltration of the normal tissue. (C) Normal bone tissue. (D) 2‐DE shows differential expression protein from A, B, C (indicated protein marked by red circle).

To explore the pathogenesis and possible biomarkers of BM, we identified more than 1300–1800 spots from 2‐DE and prioritized proteins that were significantly different in their expression between LUAD versus BM samples (Fig. 2A), bone infiltration (Fig. 2B), and normal bone (Fig. 2C) to the map matching (Fig. 2D). We selected 26 differentially expressed proteins which were then isolated, digested, and identified using matrix‐assisted laser desorption/ionization time of flight mass spectrometry (MALDI‐TOF‐MS). From these studies, we identified four proteins that appeared to be significantly associated with BM, namely, ENO1, RPLP2, CAPS1, and NMEI‐NME2 (*P* < 0.05, ratio ≥1.5). We prioritized these hits for further study. The details of these four proteins are listed in Table 3.

**Table 3:** The proteins information from 2‐DE

Group ID	Proteins information
C1170	Enolase 1 (Homo sapiens)
C1194	CAPS calcyphosine (Homo sapiens)
C2184	Ribosomal protein P2 (Homo sapiens)
C2168	NME1‐NME2 readthrough transcript (Homo sapiens)

### Kaplan–Meier analyses

**Figure 3:**
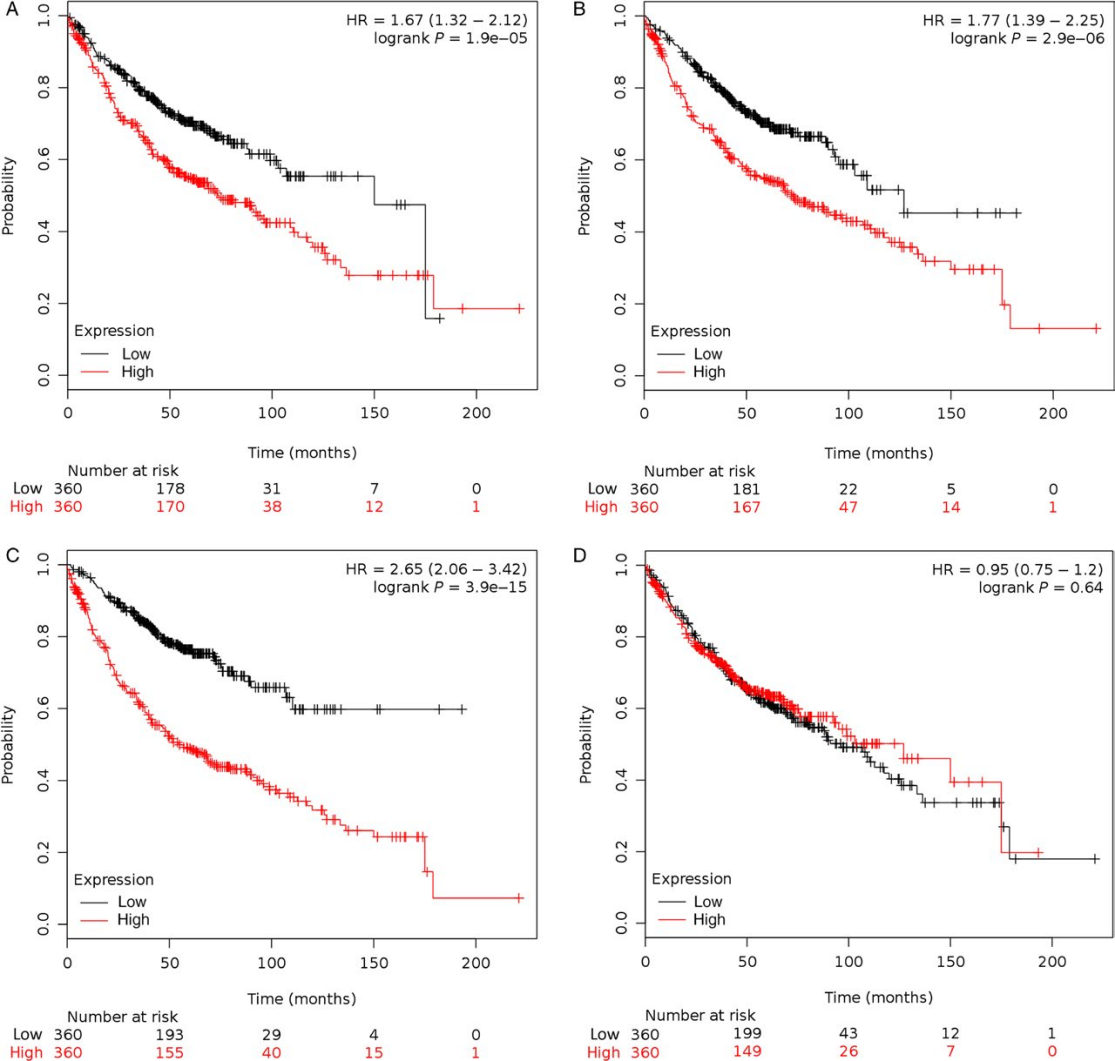
Proteomic spots from LUAD with BM were significantly associated with survival. Kaplan–Meier curves were used to analyze the association of the genes signature with clinical outcomes (A: ENO1, B: RPLP2, C: NME1‐NME2, D: CAPS1). *P *<* *0.05 was considered statistically significant.

To identify whether the expression of these four proteins was related to the patients’ survival, Kaplan–Meier plotter (KM Plotter) was used to generate patient survival curves (Fig. 3). The clinical characteristics can be found online (http://kmplot.com). In LUAD, ENO1 (HR = 1.67, 95% CI (1.32–2.12), logrank *P* = 1.9e‐05), RPLP2 (*H* = 1.77, 95% CI (1.39–2.25), logrank *P* = 2.9e‐06), and NME1‐NME2 (HR = 2.65, 95% CI (2.06–3.42), logrank *P* = 3.9e‐15) were inversely associated with better prognosis. CAPS1 (HR = 0.95, 95% CI (0.75–1.2), logrank *P* = 0.64) did not meet statistical significance.

### TCGA RNA expression levels

To verify our 2‐DE results, we used the TCGA data portal to analyze the RNA level of these proteins in lung tumors. We found that ENO1 expression was increased (adjusted *P *<* *0.05, foldchange >2), and CAPS1 expression was decreased (adjusted *P *<* *0.05, foldchange >2), while RPLP2 and NME1‐NME2 demonstrated no discrepancy (adjusted *P *>* *0.05, foldchange <2) (Table 4).

**Table 4:** The differential proteomics of LUAD with BM

ID	BaseMean	FoldChange	Log2FoldChange	*P*val	*P*adj
ENO1|2023	79109.65558	2.31367133	1.210183936	0.0004285	0.002402
RPLP2|6181	23047.9247	1.238244056	0.308295696	0.21737	0.4063166
NME1‐NME2|654364	223.6643777	1.99866556	0.999037084	0.064874	0.1625996
CASP1|828	1129.870869	0.510940442	−0.968772963	5.34E‐07	5.74E‐06

*P *<* *0.05 was considered statistically significant.

### Immunohistochemical assays

**Figure 4:**
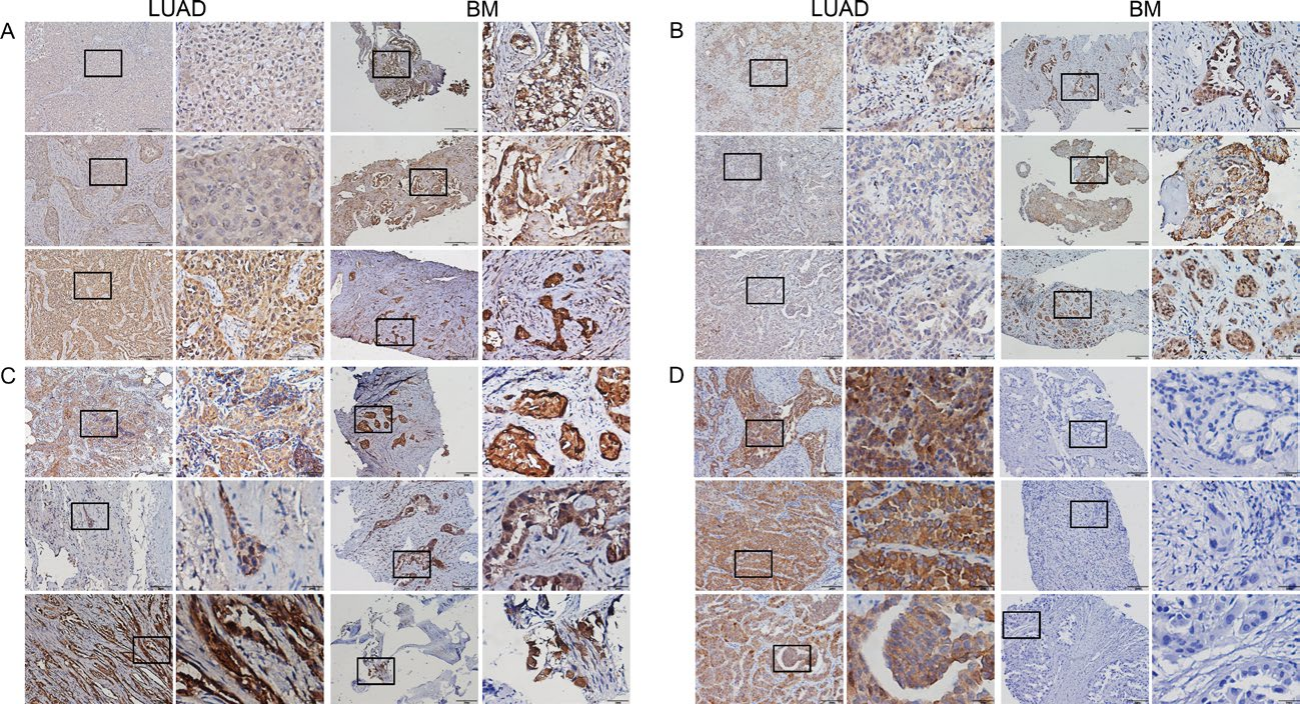
Differential expression of proteins tested by IHC. Detection of ENO1, RPLP2, NME1‐NME2, and CAPS1 in LUAD tissues compared with BM ones (original magnification x100, x400, A, B, C, and D: ENO1, RPLP2, NME1‐NME2, and CAPS1: *n* = 3 per LUAD and LUAD with BM). Representative data were shown.

Immunohistochemical studies revealed that ENO1 (OR = 1.929, 95% CI (0.915, 4.066), *P *=* *0.023), RPLP2 (OR = 0.003, 95% CI (0.294, 7.645), *P *=* *0.954), and NME1‐NME2 (OR = 0.313, 95% CI (0.033, 2.920), *P *<* *0.0001) strongly positively stained in LUAD samples with BM. CAPS1 was lightly stained in LUAD with BM (OR = 0.010, 95% CI (0.001, 0.120), *P *<* *0.0001) (Fig. 4). We can also observe that these proteins were low or even not expressed in normal bone tissue, and they almost only expressed in metastatic lung cancer cells. The IHC study also ruled out the effect of basal expression of these proteins in bone tissues.

### Overall survival and odds ratio

**Figure 5:**
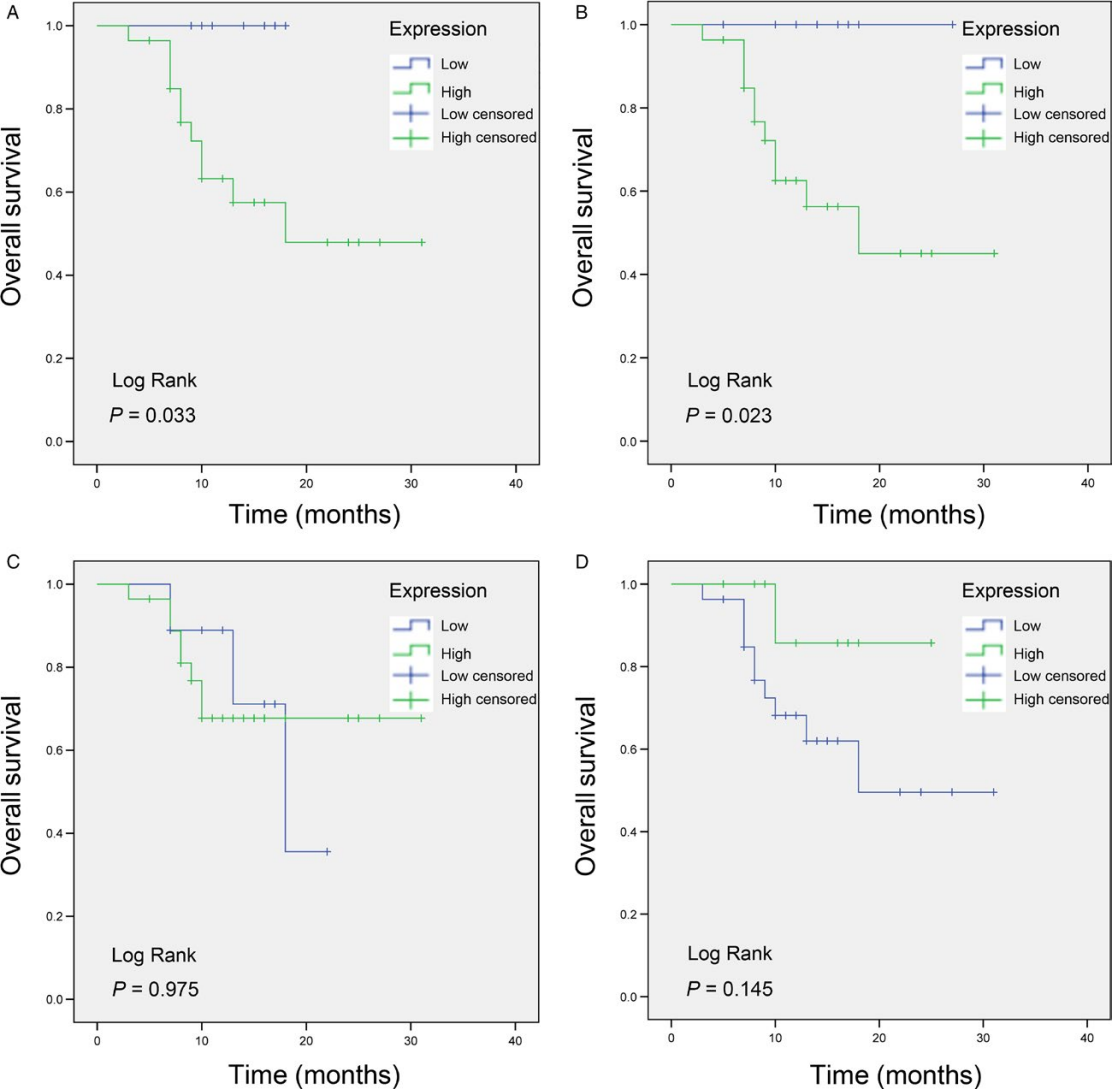
The 37 patients’ OS in the differential expression proteins. (A, B) The OS was significantly different in the differential expression level of ENO1 (*P* = 0.033) and RPLP2 (*P* = 0.023). (C, D) The OS of NME1‐NME2 (*P *=* *0.975) and CAPS1 (*P *=* *0.145) was not significantly different.

To delineate the relationship between expression of these four proteins and patient survival, 37 patients were followed up for more than 1 year. The OS was significantly different in patients with differentially expressed ENO1 (*P* = 0.033) and RPLP2 (*P* = 0.023). However, the differentially expressed NME1‐NME2 (*P *=* *0.975) and CAPS1 (*P *=* *0.145) were not significantly different. The survival curves are presented in Figure 5, and the odds ratio (OR) of these proteins between LUAD and LUAD with BM are described in Table 5.

**Table 5:** The analysis of OR of these proteins

Proteins	Group	Expression		OR	95% CI	*P*
		*H*	*L*			
ENO1	LUAD	4 (44.4%)	5 (55.6%)	7.5	1.387, 40.562	0.034
	BM	24 (85.7%)	4 (14.3%)			
RPLP2	LUAD	6 (66.7%)	3 (33.3%)	0.003	0.294, 7.645	0.954
	BM	21 (75%)	7 (25%)			
NME1‐NME2	LUAD	8 (88.9%)	1 (11.1%)	0.313	0.033, 2.920	0.538
	BM	20 (71.4%)	8 (28.6%)			
CAPS1	LUAD	8 (88.9%)	1 (11.1%)	0.01	0.001, 0.120	<0.0001
	BM	2 (7.1%)	26 (92.9%)			

*H* indicates high expression; *L*, low expression; OR, odds ratio; CI, confidence interval; LUAD, lung adenocarcinoma; BM, bone metastasis; *P *<* *0.05 was considered statistically significant.

### Western blot

**Figure 6:**
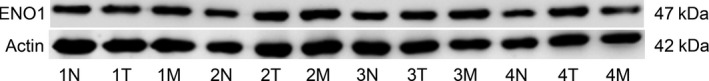
ENO1 was highest expressed in LUAD with BM, followed by LUAD samples, and poorly expressed in para‐LUAD. Four samples were analyzed in each group. Representative data were shown. N, normal; T, tumor; M, metastasis.

Western blot analysis was used to check the expression of ENO1. We found that ENO1 was most highly expressed in LUAD with BM, followed by LUAD samples, while para‐LUAD tissue demonstrated the lowest ENO1 expression (Fig. 6).

## Discussion

Thirty nine percent of patients with LUAD present with BM 12. Bae et al. found that patients with a single bone metastasis, EGFR TKI treatment, or a histology of nonsquamous cell carcinoma had good prognosis 13. The standard of care for patients with advanced‐stage cancers has shifted based on the molecular profile of the tumor 14. For example, bone markers have improved greatly and could be useful for early diagnosis of BM 15. It is increasingly critical to identify new proteins that could serve as novel biomarkers or therapeutic targets. Proteomics has been previously used in different tumors to identify novel biomarkers 16, 17, and some groups have previously used proteomics to explore BM biology 18, 19, 20. However, they all used mouse models or human cell lines for their studies.

In this study, we investigated the differential expression of proteins between traumatic amputation bone tissue, LUAD, and LUAD with BM. Using proteomic approaches, we found that ENO1, RPLP2, and NME1‐NME2 were highly expressed in BM compared to LUAD, while CAPS1 was lowest expressed in LUAD with BM than LUAD and normal bone controls. Thus, we assert that proteomics should be considered as an increasingly important part of biomedicine, which allows better insights of cancer biology and makes possible the design of novel therapeutic interventions 6.

After identifying differentially expressed proteins, we verified the differences in their expression using LUAD gene expression data in TCGA datasets. Further, we used KM Plotter, a tool that uses GEO (Affymetrix microarrays only), EGA, and TCGA data to analyze survival trends in patients with cancer 21, 22, 23. We found that the KM Plotter (*n* = 1157) has more LUAD samples than TCGA (*n* = 576). Thus, we decided to use KM Plotter to generate patient survival curves. Interestingly, we found that ENO1 and RPLP2 were significantly associated with LUAD with BM, compared with LUAD without BM.

After analyzing the data, we found that ENO1 was not only highly expressed in LUAD, but also significantly related to overall patient survival. Using Western blot analyses, we found that ENO1 was highly expressed in LUAD with BM, followed by LUAD samples, and least expressed in para‐LUAD. ENO1 is a bifunctional gene encoding both a glycolytic enzyme and a DNA‐binding protein and c‐myc‐binding protein (MBP‐1) 24. ENO1 is involved in a variety of metabolic pathways and is closely related to the tumor occurrence. ENO1 has been previously found to promote tumorigenesis and metastasis via the AMPK/mTOR pathway in colorectal cancer 25. In addition, Chen et al. reported that *Helicobacter* pyloricytotoxin‐associated gene A protein upregulated *α*‐enolase expression via Src/MEK/ERK signaling in gastric cancer 26. Song et al. 27 found that ENO1 also played important roles in glioma, and Fu et al. 28 reported that ENO1 was overexpressed in nonsmall cell lung cancer (NSCLC) and promoted glycolysis, proliferation, migration, invasion, and tumorigenesis by activating the FAK‐mediated PI3K/AKT pathway. These studies lend credence to our findings that ENO1 is significantly associated with tumorigenesis and metastasis, especially in cases of LUAD with BM.

Our data also indicated that RPLP2 was upregulated in LUAD and related to poor OS of patients with BM. Ribosomal P2 is a component of the eukaryotic 60S large ribosomal subunit, which forms a complex with other phosphoproteins (ribosomal P0 and P1 proteins) in the stalk region of the subunit 29. RPLP2 is not only important for protein synthesis but also in DNA repair 30, proliferation, apoptosis, and tumorigenesis. Some groups have found that RPLP2 was related to gynecologic tumors 31, digestive system tumors such as colon cancer, and pancreatic cancer 32. Our results are consistent with these studies, and we will continue to explore the signaling pathways associated with RPLP2.

Our research also found that, although NME1‐NME2 was highly expressed in LUAD and BM clinical samples, there was no significant difference in expression between the two groups. The Nme family, previously known as Nm23 or NDPK, is involved in various molecular processes including tumor metastasis. Moreover, some members of the family—but not all—exhibit a nucleoside diphosphate kinase (NDPK) activity 33. NME1‐NME2 is identified to be downregulated in triple‐negative breast cancers (TN) 34 and other tumors. NME1‐NME2 may play an important role in the occurrence and maintenance of the tumor, but our data suggest that this gene is unlikely to be important for metastasis.

We found that CAPS1 was downregulated and that low expression of this protein was associated with improved survival in LUAD patients with BM. CAPS1 is an EF‐hand protein involved in both Ca^2+^‐phosphatidylinositol and cyclic AMP signal cascades 35 to coordinate cellular proliferation and differentiation 36. This protein localizes to the cytosol and is expressed in numerous tissues including endocrine glands (thyroid, pancreas, adrenal, and pituitary gland) and epithelia (respiratory, digestive, and genitals) 37. Some groups have found that CAPS1 is upregulated in endometrial cancer 38 and colorectal cancer 39. This was inconsistent with our findings, perhaps because the basic expression of CAPS1 in various tissues is different and the mechanisms need to be further explored.

There are some important discoveries revealed by this study, although this work does have some limitations. First, we used TCGA mRNA data to evaluate our proteomics results. However, mRNA levels are not perfect predictors of the function of the protein coded for by a particular transcript, as mRNAs often undergo significant post‐transcriptional modifications prior to their translation. Second, our OS results were generated by KM Plotter, which contains data from mostly early‐stage patients and only a few patients with BM. Third, while we identified differentially expressed proteins associated with survival, we did not explore the exact mechanism of these proteins in the process of metastasis. It is necessary to verify the mechanism of BM in mouse models 40. Lastly, the sample size of our study is small. However, the clinical samples we enrolled in were of significant statistical significance. Our team has significant interest in the study of BM, and the development of our clinical database is an important contribution to this field. Thus, we will pursue more in‐depth exploration as to why these proteins are highly expressed in BM and what their contribution to BM pathogenesis is.

In summary, we report here for the first time using human LUAD with BM tissues to identify ENO1, CAPS1, RPLP2, and NME1‐NME2 as significantly associated with BM occurrence and patient's survival. The study provides new targets for drug development and disease biomarkers of LUAD with BM.

## Conflict of Interest

None declared.
